# A case report of thrombocytopenic COVID-19 and Miller–Fisher syndrome on a concurrent chronic immune neuropathy

**DOI:** 10.1097/MD.0000000000038304

**Published:** 2024-05-24

**Authors:** Lisle Blackbourn, Umair Hamid, Janaki Tokala, Gregory Blume

**Affiliations:** a Department of Neurology, University of Illinois College of Medicine Peoria, Peoria, IL, USA; b OSF Illinois Neurological Institute, Peoria, IL, USA.

**Keywords:** acute inflammatory demyelinating polyneuropathy, chronic inflammatory demyelinating polyneuropathy, COVID-19, Guillain–Barre syndrome, Miller–Fisher syndrome

## Abstract

**Rationale:**

Miller–Fisher syndrome (MFS) is a rare subtype of Guillain–Barre syndrome with classic features of ataxia, areflexia, and ophthalmoplegia that can be caused by a preceding infection including COVID-19. We present a current, asymptomatic thrombocytopenic COVID-19 infection as a cause of MFS in a 60-year-old male with a concurrent chronic immune neuropathy.

**Patient concerns:**

A 60-year-old male presenting with acute symptoms of MFS including ataxia, areflexia, and ophthalmoplegia on a chronic immune neuropathy for at least 1 year and concurrent asymptomatic COVID-19 positive infection.

**Diagnosis:**

MFS suspected secondary to a current thrombocytopenic COVID-19 infection.

**Interventions:**

Five days of intravenous immune globulin with continued monthly intravenous immune globulin as an outpatient, follow-up long-term in a neuromuscular clinic, electromyography as an outpatient, and continued physical therapy.

**Outcomes:**

The patient significantly improved after initial treatment.

**Lessons:**

The full effect of COVID-19 on the various Guillain–Barre syndrome subtypes is unknown, although it clearly can be a cause of the various variants including being caused by a current, asymptomatic infection.

## 1. Introduction

Guillain–Barre syndrome (GBS) typically presents with progressive symmetric weakness in the lower extremities with areflexia. Acute inflammatory demyelinating polyneuropathy is the most common form of GBS. Other variants include acute motor axonal neuropathy (AMAN), acute motor and sensory motor neuropathy (AMSAN), and Miller–Fisher syndrome (MFS).^[1,2]^ Typically, cases present within 4 weeks after an infection, with most cases presenting after 2 weeks. GBS is a clinical diagnosis. Investigations such as CSF analysis, serum antibodies, nerve conduction studies, and imaging can help support a GBS diagnosis or rule out other causes. For example, GBS CSF analysis can show a cytoalbuminologic dissociation, meaning a normal cell count with increased protein seen in the CSF.

GBS is believed to be the result of an abnormal autoimmune response from a preceding infection through molecular mimicry driving an inflammatory response in the body. MFS is a rare subtype of GBS.^[3]^ MFS can present with ataxia, areflexia, and ophthalmoplegia due to 3rd, 4th, and 6th cranial nerve dysfunction. GQ1b ganglioside antibodies are a typical serological finding and can be seen in about 80% of cases.^[4,5]^

While many different preceding infections have been shown to cause GBS and MFS, COVID-19 has also been linked to cause GBS and MFS.^[6–9]^ Typically, GBS and MFS have developed about 2 weeks after COVID-19 infection.^[7,8]^

Chronic inflammatory demyelinating polyneuropathy (CIDP) is an immune-mediated motor neuropathy with symmetrical progressive weakness of both the proximal and distal muscles.^[10]^ It can be treated with IVIg due to it being an immune motor neuropathy, however, some patients do not require any treatment due to how mild symptoms can present. Electrodiagnostic evidence such as slowed motor conduction velocities or motor conduction block in the appropriate clinical scenario are necessary to make the diagnosis of CIDP.

Here we present a case of a 60-year-old male presenting with acute symptoms of MFS including ataxia, areflexia, and ophthalmoplegia on a chronic immune neuropathy for at least 1 year and concurrent asymptomatic COVID-19 positive infection.

## 2. Case details

A 60-year-old male was in his usual state of health before going to bed the previous night. The next day, the patient awoke about 13 hours later and noticed slurred speech, generalized weakness, double vision, difficulty swallowing, and a swollen tongue. The patient went to a nearby health center where he was found to have generalized weakness. The patient was transferred to our hospital for further diagnosis and management. He had a past medical history of restless leg syndrome and a neck abscess treated completely 2 weeks prior to presentation.

On physical exam, the patient had significant speech ataxia, hypophonic speech, and intact naming and comprehension with impaired fluency most likely due to dysarthria. Ophthalmoplegia was seen with bilateral medial rectus weakness, but no nystagmus. Pupils were equal, round, and reactive to light.

The patient had bilateral facial weakness and was unable to whistle or puff his cheeks out. He was noted to have 4/5 power in the bilateral deltoids, grip strength, knee flexion and extension, and left bicep. A 3/5 power was seen in the right bicep and bilateral hip flexion. No sensation changes were seen in light touch or sharp touch. Vibration was reduced in all extremities and proprioception was reduced in the bilateral lower extremities.

There were no reflexes elicited in the patellar and ankle bilaterally, +1 biceps and triceps on the left upper with absence on the right, and +1 in the brachioradialis bilaterally. The next day, the patient became areflexic in all but the bilateral brachioradialis where he remained +1.

Finger-to-nose showed extreme ataxia in both upper extremities. The patient had ataxia in bilaterally heel-to-shin testing and exhibited truncal ataxia. Gait was unable to be accessed.

There also was bilateral hypothenar eminence, bilateral thenar eminence, bilateral interossei, and bilateral quadriceps atrophy. The patient stated that this muscle wasting began about 1 year prior to presentation to the hospital. Fasciculations were also seen in the bilateral quadriceps.

Examination of the right-lower and left-lower lung fields revealed decreased breath sounds. The patient’s negative inspiratory force (NIF) was −5 at his best attempt.

A complete metabolic panel, magnesium level, vitamin levels, IgA level, and BNP were all normal on admission. A complete blood count was normal except for a low platelet level of 77k.

MRI brain and MRA showed no acute intracranial abnormality with no large vessel stenosis. MRI C-spine with and without contrast showed multilevel degenerative changes in the cervical spine with stenosis at C5 to C6.

The patient was screened for COVID-19 via PCR testing per hospital policy for all patients being admitted and resulted positive. The patient denied any fevers, cough, shortness of breath, sore throat, chest pain, or any other signs of COVID-19 illness. Further workup for infection was negative.

Given the onset and progression of symptoms and exam findings of ataxia, weakness, areflexia, and facial weakness in the setting of COVID, there was concern for possible MFS variant of GBS. It was decided to start IVIg for 5 days, obtain NIF values every 8 hours to monitor respiratory status, and obtain ganglioside antibodies.

Over the next couple of days, the patient’s laboratory values remained steady. His platelets, however, dropped gradually to a low of 54k, before rising to 121k prior to discharge. Ganglioside panel showed high levels of GM1, GD1a, GD1b, and GQ1b antibodies. Levels of ASIALO-GM1 and GM2 were normal. Please see Table 1 for further information.

**Table 1:** Ganglioside antibody panel results of patient.

Antibody	Level	Reference range (Units IV)
ASIALO-GM1	15	0 to 50
GM1	128	0 to 50
GM2	10	0 to 50
GD1a	81	0 to 50
GD1B	125	0 to 50
GQ1B	56	0 to 50

After 5 doses of IVIg, the patient significantly improved. Speech and swallowing returned to baseline. The ophthalmoplegia and bilateral medial rectus weakness resolved. Muscle strength greatly improved bilaterally in the upper and lower extremities. The truncal ataxia also resolved. Finger-to-nose and heel-to-shin displayed bilateral improvement but remained ataxic. High-dose thiamin was added in hopes it would help with nerve healing. The patient was still areflexic with +1 bilateral brachioradialis reflexes. The patient also had improved and stable NIF scores of −40. The patient’s fasciculations were still seen after treatment.

It was planned to continue monthly IVIg as an outpatient, follow-up long-term in a neuromuscular clinic, obtain an EMG as an outpatient, and continue physical therapy.

In summary, we present a 60-year-old male presenting with acute symptoms of MFS including ataxia, areflexia, and ophthalmoplegia on a chronic immune neuropathy for at least 1 year. The patient had a ganglioside panel that showed high levels of GM1, GD1a, GD1b, and GQ1b antibodies and had significant improvement in the treatment for MFS. The patient also had a concurrent asymptomatic COVID-19 causing thrombocytopenia.

## 3. Discussion

The patient in this case has the typical clinical features of MFS and improvement with proper treatment. The concurrent asymptomatic infection of COVID-19 is unique among cases of MFS. There have been reported cases of GBS that develop COVID-19 symptoms shortly after GBS symptoms and that developed GBS with concurrent asymptomatic COVID-19.^[7]^ However, the few reported MFS related to COVID-19 averages 14.75 days after COVID-19 onset according to one systematic review.^[8]^ One case report did present a patient with symptomatic COVID-19 one day prior to onset of MFS symptoms.^[10]^ The patient did report a neck abscess 2 weeks prior to symptom onset, which could very likely be the trigger for his MFS, however, given reported concurrent COVID-19 cases causing GBS and MFS, it cannot be ruled out as the potential cause for his presentation.

Interestingly, thrombocytopenia has been shown in meta-analyses to be associated with more severe COVID-19 infections and high mortality among those patients.^[11]^ The patient presented here, while having a low baseline platelet count prior to this admission, did exhibit a drop in platelets that we attributed to his COVID-19 infection. This being his only laboratory or clinical finding related to his COVID-19 infection. This subsequently returned to his baseline after treatment.

**Figure 1.**
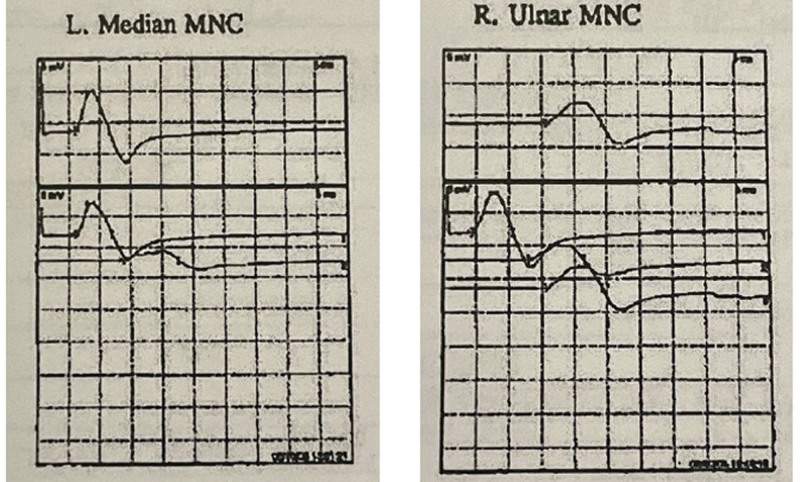
Parts of a past motor nerve conduction test showing conduction block in patient.

The patient also presented with atrophy of the bilateral hypothenar eminence, thenar eminence, interossei, and quadriceps compatible with a chronic neuropathy process. Although anti-GM1 antibodies can be present in GBS/MFS, the elevation in this patient could also be explained by an underlying demyelinating motor neuropathy given the clinical examination findings mentioned above.^[12]^ Further, the patient had past motor nerve conduction tests showing conduction block as far back as 2005 as seen in Figure 1. For this reason, continued monthly treatment was chosen for the patient. It also should be noted that the patient’s baseline reflexes are unknown due to the underlying chronic neuropathy.

Previously, the patient’s neuropathy had been deemed due to alcohol use. Motor conduction block, however, is atypical for neuropathy associated solely with alcohol intake.

There is one case report of a patient with a 6-year history of a CIDP exhibiting fluctuating symptoms of ophthalmoplegia, ataxia, and areflexia (MFS) with high levels of GQ1b antibodies.^[13]^ No case reports were found of multifocal motor neuropathy and MFS in our search. Monthly IVIg will hopefully mute any further inflammatory process in our patient from his MFS and chronic neuropathy.

An outpatient EMG is planned for this patient. It will not be able to differentiate between the MFS and a chronic neuropathy but will help to rule out amyotrophic lateral sclerosis (ALS) and similar disorders. Interestingly, ganglioside antibodies, usually GM1, have been seen in ALS, with one case having been reported where ALS was diagnosed in a patient who had recovered from MFS.^[14]^ One question that remains in our patient is why he still has +1 brachioradialis reflexes. One theory is that he was possibly hyperreflexic from an underlying myelopathy due to his cervical stenosis. The patient’s remaining ataxia could also be due to his high GD1b antibody level, which has been shown to be higher in ataxia GBS than non-ataxic GBS.^[15]^

As previously discussed, the patient’s neck abscess 2 weeks prior to symptom onset could very likely be the trigger for his MFS, confounding our belief in the concurrent COVID-19 infection as the triggering event. Another limitation is an EMG will not be able to differentiate between the MFS and a chronic neuropathy. Unfortunately, our patient passed away of an unrelated disease prior to obtaining an EMG.

## 4. Conclusion

Prompt recognition and treatment in GBS and MFS are essential. The full effect of COVID-19 on the various GBS subtypes is unknown, although it clearly can be a cause of the various variants. Follow-up in patients is important to help reduce any post-complications or residual effects in such syndromes.

## Author contributions

**Conceptualization:** Lisle Blackbourn, Umair Hamid, Janaki Tokala, Gregory Blume.

**Writing – original draft:** Lisle Blackbourn, Umair Hamid, Janaki Tokala, Gregory Blume.

**Writing – review & editing:** Lisle Blackbourn, Umair Hamid, Janaki Tokala, Gregory Blume.

**Supervision:** Gregory Blume.
